# Soluble suppression of tumorigenicity 2 (sST2) for predicting disease severity or mortality outcomes in cardiovascular diseases: A systematic review and *meta*-analysis

**DOI:** 10.1016/j.ijcha.2021.100887

**Published:** 2021-10-18

**Authors:** Christina Ip, King Sum Luk, Vincent Lok Cheung Yuen, Lorraine Chiang, Ching Ki Chan, Kevin Ho, Mengqi Gong, Teddy Tai Loy Lee, Keith Sai Kit Leung, Leonardo Roever, George Bazoukis, Konstantinos Lampropoulos, Ka Hou Christien Li, Gary Tse, Tong Liu, International Health Informatics Study (IHIS) Network

**Affiliations:** a Epidemiology Research Unit, Cardiovascular Analytics Group, Hong Kong, China-UK Collaboration; b Tianjin Key Laboratory of Ionic-Molecular Function of Cardiovascular disease, Department of Cardiology, Tianjin Institute of Cardiology, Second Hospital of Tianjin Medical University, Tianjin 300211, China; c Emergency Medicine Unit, Li Ka Shing Faculty of Medicine, The University of Hong Kong, Hong Kong, China; d Department of Clinical Research, Federal University of Uberlândia, Uberlândia, MG, Brazil; e Second Department of Cardiology, Evangelismos General Hospital of Athens, Athens, Greece; f Faculty of Medicine, Newcastle University, Newcastle, UK; g Kent and Medway Medical School, Canterbury, United Kingdom

**Keywords:** Soluble suppression of tumorigenicity 2, sST2, Severity, Mortality, Heart failure, Coronary artery disease

## Abstract

**Objectives:**

Soluble suppression of tumorigenicity 2 (sST2) is a member of the interleukin-1 receptor family. It is raised in various cardiovascular diseases, but its value in predicting disease severity or mortality outcomes has been controversial. Therefore, we conducted a systematic review and *meta*-analysis to determine whether sST2 levels differed between survivors and non-survivors of patients with cardiovascular diseases, and whether elevated sST2 levels correlated with adverse outcomes.

**Methods:**

PubMed and Embase were searched until 23rd June 2021 for studies that evaluated the relationship between sST2 levels and cardiovascular disease severity or mortality.

**Results:**

A total of 707 entries were retrieved from both databases, of which 14 studies were included in the final *meta*-analysis. In acute heart failure, sST2 levels did not differ between survivors and non-survivors (mean difference [MD]: 24.2 ± 13.0 ng/ml; P = 0.06; *I*^2^: 95%). Elevated sST2 levels tend to be associated with increased mortality risk (hazard ratio [HR]: 1.12, 95 %CI: 0.99–1.27, P = 0.07; *I*^2^: 88%). In chronic heart failure*,* sST2 levels were higher in non-survivors than in survivors (MD: 0.19 ± 0.04 ng/ml; P = 0.001; *I*^2^: 0%) and elevated levels were associated with increased mortality risk (HR: 1.64, 95% CI: 1.27–2.12, P < 0.001; *I*^2^: 82%). sST2 levels were significantly higher in severe disease compared to less severe disease (MD: 1.56 ± 0.46 ng/ml; P = 0.001; *I*^2^: 98%). Finally, in stable coronary artery disease, sST2 levels were higher in non-survivors than survivors (MD: 3.0 ± 1.1 ng/ml; P = 0.005; *I*^2^: 80%) and elevated levels were significantly associated with increased mortality risk (HR: 1.32, 95% CI: 1.04–1.68, P < 0.05; *I*^2^: 57%).

**Conclusions:**

sST2 significantly predicts disease severity and mortality in cardiovascular disease and is a good predictor of mortality in patients with stable coronary artery disease and chronic heart failure.

## 1 Introduction

The interleukin (IL)-1 receptor family is a family of receptors mediating the activities of specific members of the IL-1 family of ligands, which are important in both innate and adaptive immune response [1]. Suppression of Tumorigenicity 2 (ST2) is a member of the IL-1 receptor family and it consists of two important isoforms, namely, ST2 ligand (ST2L) and soluble ST2 (sST2) [2]. ST2L is a transmembrane receptor while sST2 is a soluble receptor that circulates in the bloodstream [2]. IL-33 is a functional ligand of ST2L receptor [3]. Like other ligands in the IL-1 family, the binding of IL-33 and ST2L on the inflammatory cell membrane activates subsequent intracellular signaling and mediates its pro-inflammatory action [4].

Apart from the role of ST2 in mediating inflammatory responses, the expression of ST2 is stimulated by cardiomyocyte stretch [5]. Mouse studies have shown that mice lacking the ST2 gene had increased cardiac fibrosis and cardiomyocyte cross-sectional area after transverse aortic constriction compared to mice with the ST2 gene [6]. The binding of IL-33 to ST2L exerts a protective effect against angiotensin II-driven adverse remodeling of the myocardium. sST2 acts as a decoy receptor and binds to IL-33, which reduces the amount of IL-33 available for interacting with ST2L [7]. Hence, the cardioprotective action of IL-33 is reduced when circulating sST2 levels are elevated [6].

Based on the above principles, sST2 has been widely investigated on its potential to become a prognostic biomarker for pulmonary hypertension [8], post-aortic valve replacement [9] and cardiovascular diseases (CVDs) [10]. Although some studies reported its predictive value for risk stratification in the context of CVDs, data on its value in predicting disease severity or mortality outcomes are inconclusive. Therefore, we conducted a systematic review and *meta*-analysis on the utility of sST2 in predicting disease severity or mortality outcomes in cardiovascular diseases, including acute heart failure, chronic heart failure, stable acute coronary syndrome and chest pain.

## 2 Methods

### 2.1 Search strategy, inclusion and exclusion criteria

This systematic review and *meta*-analysis was performed and reported according to the Preferred Reporting Items for Systematic Reviews and Meta-Analyses (PRISMA) statement. PubMed and EMBASE were searched for studies that investigated the relationship between soluble suppression of tumorigenicity-2 (sST2) levels and cardiovascular diseases using the following terms: [(Soluble suppression of tumorigenicity-2 OR sST2) and (severity OR mortality OR outcome)]. The databases were searched from inception to 23rd June 2021, with no language restrictions. The following inclusion criteria were applied: i) case-control, prospective or retrospective cohort studies in humans; ii) sST2 values were provided and related to disease severity or mortality in cardiovascular diseases; iii) the study assessed cardiovascular diseases. The following studies were excluded: i) did not assess cardiovascular diseases; ii) systematic reviews, *meta*-analyses and editorials; iii) absence of comparable data.

Quality assessment was performed using the Newcastle–Ottawa Quality Assessment Scale (NOS). The point score system evaluated the categories of 1) study participant selection, 2) comparability of the results, and 3) quality of the outcomes. The following characteristics were assessed: a) representativeness of the exposed cohort; b) selection of the non-exposed cohort; c) ascertainment of exposure; d) demonstration that outcome of interest was not present at the start of study; e) comparability of cohorts on the basis of the design or analysis; f) assessment of outcomes; g) follow-up period sufficiently long for outcomes to occur; and h) adequacy of follow-up of cohorts. Each criteria met contributed to a point in the scale, which varied from zero to nine points: Study quality was deemed poor if<5 points, fair if 5 to 7 points, and optimal if 8 or more points. The details of the NOS quality assessment are shown in **Supplementary Table 1**.

### 2.2 Data extraction and statistical analysis

Data extraction was performed with a pre-specified spreadsheet in Microsoft Excel. All publications identified were assessed for compliance with the inclusion criteria. In this *meta*-analysis, the extracted data elements consisted of: surname of first author, publication year, study design, follow-up duration, sample size, gender, age, and cut-off point for sST2 levels. Two reviewers (CI and KSL) independently reviewed each included study and disagreements were resolved by adjudication with input from a third reviewer (GT).

Mean differences in sST2 levels between survivors and non-survivors were extracted from each study and subsequently pooled in our *meta*-analysis. For the relationship between sST2 and mortality, multivariate adjusted hazard ratios (HR) with 95% confidence interval (CI) were extracted and analyzed for each study. When values from multivariate analysis were not available, those from univariate analysis were used. When the latter were not provided, raw data where available were used to calculate unadjusted risk estimates.

Heterogeneity across studies was determined using Cochran's Q value, which is the weighted sum of squared differences between individual study effects and the pooled effect across studies; and the *I^2^* statistic from the standard chi-square test, which describes the percentage of the variability in the effect estimates resulting from heterogeneity. A value of *I^2^* > 50% was considered to reflect significant statistical heterogeneity. The random-effects model using the inverse variance heterogeneity method was used if *I^2^* > 50%. To locate sources of heterogeneity, sensitivity analysis was performed by excluding one study at a time. Subgroup analyses based on different disease conditions and different endpoints were performed. Funnel plots showing standard errors or precision against the logarithms of the odds ratio were constructed. The Begg and Mazumdar rank correlation test and Egger’s test were used to assess for possible publication bias.

## 3 Results

**Fig. 1:**
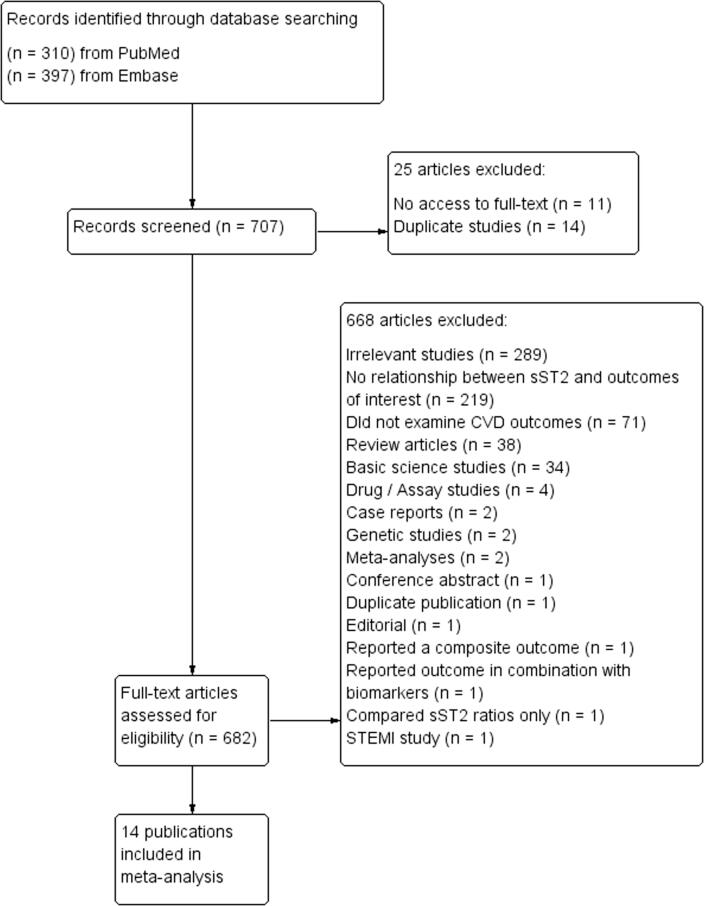
PRISMA flow chart of the study selection process.

The flow diagram detailing the search strategy and study selection process is shown in Fig. 1. A total of 310 and 397 entries were retrieved from PubMed and Embase, of which 14 studies were included in the final *meta*-analysis [11], [12], [13], [14], [15], [16], [17], [18], [19], [20], [21], [22], [23], [24]. The baseline characteristics of included studies were listed in Table 1. All studies were prospective in design. The predictive value of sST2 was examined in the following conditions: acute heart failure (n = 5), chronic heart failure (n = 6) and stable coronary artery disease (n = 3).

**Table 1:** Characteristics of the studies included in this *meta*-analysis.

**First author / Year**	**Population**	**Country**	**Method of sST2 detection**	**sST2 cut-off value (ng/ml)**	**Sample size (n)**	**Mean age (years)**	**Males (n[%])**	**Follow-up (months)**	**Ref**
Jin, 2017	Acute heart failure	China	Enzyme-linked immunosorbent assay (ELISA) (R&D Systems, Abingdon, UK)	36	287	60.5	181 (63.1%)	12	[11]
Manzano-Fernández, 2012	Acute heart failure	Spain	Presage → ST2 (Critical Diagnostics, USA)	76	72	69	47 (65.3%)	25	[12]
Manzano-Fernández, 2011	Acute heart failure	United States, Austria, and Spain	ELISA (Medical and Biological Laboratories, USA)	0.53	447	73	290 (64.9%)	12	[13]
Pascual-Figal, 2011	Acute heart failure	Spain	Presage → ST2 (Critical Diagnostics, USA)	65	107	72	47 (43.9%)	25	[14]
Mueller, 2008	Acute heart failure	Austria	BEP® 2000 instrument (Dade Behring); sandwich double monoclonal antibody ELISA (Medical and Biological Laboratories, USA)	700	137	–	–	–	[15]
Sinning, 2017	Chronic heart failure	Germany	Presage → ST2 (Critical Diagnostics, San Diego, California)	–	5000	56	2540 (50.8%)	88	[18]
Gül, 2017	Chronic heart failure	Turkey	Presage → ST2 Assay (Critical Diagnostics, USA)	30	130	67	90 (69.2%)	12	[16]
Wojtczak-Soska, 2014	Chronic heart failure	Poland	Sandwich ELISA kit (Medical and Biological Laboratories, Japan)	0.34	167	63	139 (83.2%)	12	[20]
Sobczak, 2014	Chronic heart failure	Poland	Sandwich monoclonal ELISA kits (Medical and Biological Laboratories, USA)	0.30	145	62	120 (82.8%)	12	[21]
Zhang, 2014	Chronic heart failure	China	ELISA in a microtiter plate format (Critical Diagnostics, USA)	–	1528	58	1075 (70.4%)	8	[19]
Scott, 2011	Chronic heart failure	United Kingdom	ELISA and methylacridinium ester- labelled streptavidin on an MLX plate luminometer (Dynex Technologies Ltd, UK)	–	156	69	132 (84.6%)	15	[17]
Pfetsch, 2017	Stable coronary artery disease	Germany	ELISA (Critical Diagnostics, USA)	35	1081	59	915 (84.6%)	156	[22]
Dieplinger, 2014	Stable coronary artery disease	Germany	BEP® 2000 instrument (Siemens Healthcare Diagnostics) with the PresageΦST2 sandwich immunoassay assay (Critical Diagnostics, USA)	25	1345	65	1008 (74.9%)	118	[24]
Demyanets, 2014	Stable coronary artery disease	Austria	ELISA (R&D Systems, USA)	–	373	64	279 (74.8%)	43	[23]

### 3.1 sST2 and mortality in acute heart failure

**Fig. 2:**
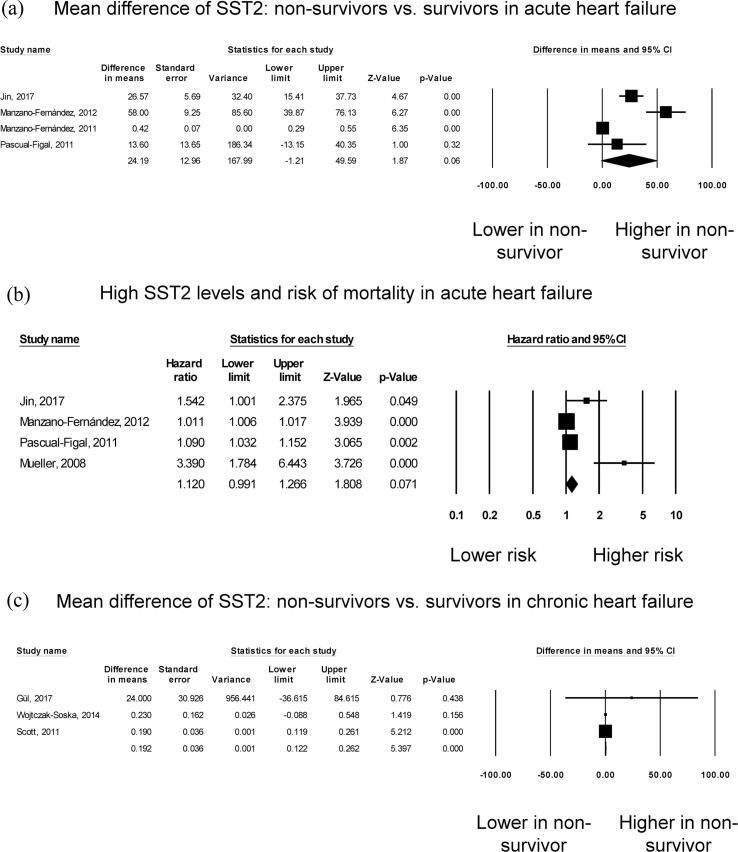

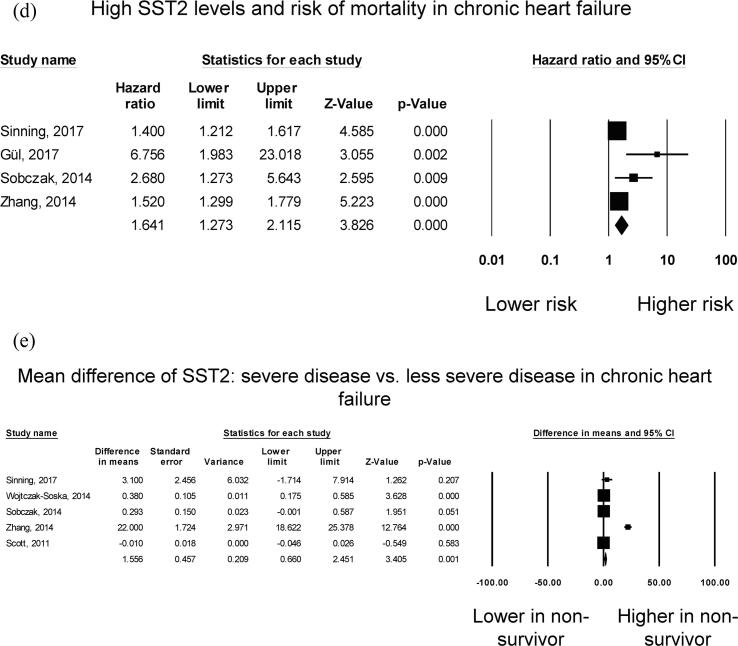
Mean difference in sST2 between non-survivors and survivors in acute heart failure (A). High sST2 and mortality risk in acute heart failure (B). Mean difference in sST2 between non-survivors and survivors in chronic heart failure (C). High sST2 and mortality risk in acute heart failure (D). Mean difference in sST2 between severe and non-severe disease in chronic heart failure (E).

A total of 1050 patients (62% male, mean age 68 years; mean follow-up duration of 14 months) from five studies assessing acute heart failure were analysed [11], [12], [13], [14], [15]. Five studies compared sST2 levels between survivors and non-survivors in acute heart failure, but one study was excluded from the *meta*-analysis because it did not provide any measure of dispersion [15]. Three studies reported significantly higher sST2 levels in non-survivors than in survivors, whereas one study found no significant difference (Fig. 2**A**). Pooled *meta*-analysis showed a mean difference of 24.2 ng/ml (standard error: 13.0 ng/ml) but this did not quite reach statistical significance (P = 0.06). *I*^2^ took a value of 95%, indicating the presence of substantial heterogeneity. The funnel plot of standard error against mean difference is shown in Supplementary Figure 1**.** Begg and Mazumdar rank correlation suggested no significant publication bias (Kendal’s Tau value 2.0, P > 0.05). Egger’s test demonstrated no significant asymmetry (intercept 4.0, t-value 2.5; *P* > 0.05). Sensitivity analysis excluding one study at a time did not significantly affect the pooled estimate (Supplementary Figure 2).

Four studies examined the risk of mortality with increased sST2 in acute heart failure (Fig. 2**B**). Elevated levels of sST2 increased the risk of all-cause mortality by 12% but this value was not statistically significant (HR: 1.12, 95% CI: 0.99 to 1.26, p = 0.07). *I*^2^ took a value of 88%, indicating substantial heterogeneity. The funnel plot is shown in Supplementary Figure 3. Begg and Mazumdar rank correlation suggested no significant publication bias (Kendal’s Tau value 0.4, P > 0.05). Egger’s test demonstrated significant asymmetry (intercept 3.6, t-value 4.6; *P* < 0.05). Sensitivity analysis excluding one study at a time did not significantly affect the pooled estimate (Supplementary Figure 4).

### 3.2 sST2 and mortality in chronic heart failure

A total of 7126 patients (57% male, mean age 57 years; mean follow-up duration of 64 months) from six studies for chronic heart failure were analysed [16], [17], [18], [19], [20], [21]. Three studies compared sST2 levels between survivors and non-survivors in chronic heart failure. Of these, one study demonstrated significantly higher sST2 levels in non-survivors than in survivors, whereas the other two studies found no significant difference (Fig. 2**C**). Nevertheless, pooled results showed a mean difference of 0.19 ng/ml (standard error: 0.04 ng/ml; P < 0.001). *I*^2^ took a value of 0%, indicating absence of heterogeneity. The funnel plot of standard error against mean difference is shown in Supplementary Figure 5. Begg and Mazumdar rank correlation suggested no significant publication bias (Kendal’s Tau value 1.0, P > 0.05). Egger’s test demonstrated no significant asymmetry (intercept 0.6, t-value 2.8; *P* > 0.05). Sensitivity analysis excluding one study at a time did not significantly affect the pooled estimate (Supplementary Figure 6).

Four studies examined the risk of mortality with increased sST2 in chronic heart failure, all of which reported significant associations (Fig. 2**D**). Our *meta*-analysis showed that elevated levels of sST2 significantly increased the risk of all-cause mortality by 64% (HR: 1.64, 95% CI: 1.27 to 2.12, p < 0.001). *I*^2^ took a value of 82%, indicating the presence of substantial heterogeneity. The funnel plot for standard error against mean difference is shown in Supplementary Figure 7. Begg and Mazumdar rank correlation suggested significant publication bias (Kendal’s Tau value 1.0, P < 0.05). Egger’s test demonstrated significant asymmetry (intercept 3.0, t-value 6.1; *P* < 0.01). Sensitivity analysis excluding one study at a time did not significantly affect the pooled estimate (Supplementary Figure 8).

Five studies examined the relationship between sST2 levels and disease severity in chronic heart failure. Of these, four studies reported significantly higher sST2 levels in severe disease than in less severe disease, whereas the remaining study found no significant difference (Fig. 2**E**). Nevertheless, pooled *meta*-analysis showed a mean difference of 1.56 ng/ml (standard error: 0.46 ng/ml; P = 0.001). *I*^2^ took a value of 98%, indicating the presence of substantial heterogeneity. The funnel plot of standard error against mean difference is shown in Supplementary Figure 9. Begg and Mazumdar rank correlation suggested no significant publication bias (Kendal’s Tau value 0.2, P > 0.05). Egger’s test demonstrated no significant asymmetry (intercept 5.5, t-value 2.0; *P* > 0.05). Sensitivity analysis excluding one study at a time did not significantly affect the pooled estimate (Supplementary Figure 10).

### 3.3 sST2 and mortality in stable coronary artery disease

**Fig. 3:**
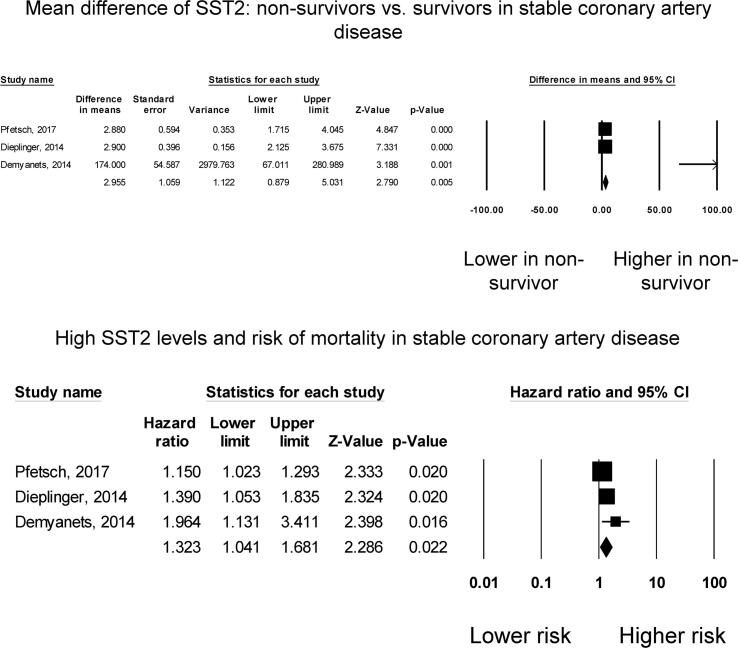
Mean difference in sST2 between non-survivors and survivors in stable coronary artery disease (A). High sST2 and mortality risk in stable coronary artery disease (B). Mean difference in sST2 between non-survivors and survivors in patients with dyspnoea or chest pain (C). High sST2 and mortality risk in patients with dyspnoea or chest pain (D).

A total of 2799 patients (79% male, mean age 63 years; mean follow-up duration of 122 months) from three studies on stable coronary artery disease were included [22], [23], [24]. Three studies compared sST2 levels between survivors and non-survivors in stable coronary artery disease. All reported significantly higher sST2 levels in non-survivors than in survivors (Fig. 3**A**). Pooled *meta*-analysis showed a mean difference of 2.96 ng/ml (standard error: 1.06 ng/ml; P = 0.005). *I*^2^ took a value of 80%, indicating the presence of substantial heterogeneity. A funnel plot of standard error against mean difference is shown in Supplementary Figure 11. Begg and Mazumdar rank correlation suggested no significant publication bias (Kendal’s Tau value 0.3, P > 0.05). Egger’s test demonstrated no significant asymmetry (intercept 2.9, t-value 3.4; *P* > 0.05). Sensitivity analysis excluding one study at a time did not significantly affect the pooled estimate (Supplementary Figure 12).

Three studies examined the relationship between mortality risk with sST2 levels in stable coronary artery disease, all of which demonstrated significant associations (Fig. 3**B**). Our *meta*-analysis showed that elevated levels of sST2 significantly increased the risk of all-cause mortality by 32% (HR: 1.32, 95% CI: 1.04 to 1.68, p < 0.05). *I*^2^ took a value of 57%, indicating the presence of moderate heterogeneity. The funnel plot is shown in **Supplementary Figures 13**. Begg and Mazumdar rank correlation suggested no significant publication bias (Kendal’s Tau value 1.0, P > 0.05). Egger’s test demonstrated significant asymmetry (intercept 2.4, t-value 48; *P* < 0.05). Sensitivity analysis excluding one study at a time did not significantly affect the pooled estimate (Supplementary Figure 14).

## 4 Discussion

The main findings of this systematic review and *meta*-analysis are that elevated levels of sST2 significantly predicted the severity of chronic heart failure and the risk of mortality in chronic heart failure (HR: 1.64, 95% CI: 1.27 to 2.12, p < 0.001) and stable coronary artery disease (HR: 1.32, 95% CI: 1.04 to 1.68, p < 0.05).

Novel biomarkers have emerged and played an important role in assessing and monitoring the risk of patients with cardiovascular events in recent years. For example, natriuretic peptides (NPs) are produced in response to myocardial stress; mid-regional pro-adrenomedullin (MR-proADM) is related to global stress; and ST2 reflects ventricular fibrosis and remodeling [25]. Moreover, cancer antigen-125 has been identified as a promising marker for predicting fluid overload and guiding heart failure treatment as well as predicting atrial fibrillation risk [26], [27]. Thus, biomarkers have been developed as a tool to provide additional clinical information for assessing the progression and prognosis of heart failure and other cardiovascular diseases.

Circulating sST2 levels are increased in response to inflammatory diseases and heart diseases [28]. In a mouse study, it was shown that sST2 was released in cultured myocytes upon mechanical stress and increased in blood concentration following myocardial infarction [5]. sST2 is expressed on macrovascular and microvascular endothelial cells [29] and secreted by cardiomyocytes and fibroblasts when under mechanical stress [30]. Cytokines from damaged tissues are believed to activate neighboring cells to produce sST2, which in turn prevents an uncontrolled inflammatory response [31].

The cardioprotective IL-33/ST2L signaling pathway prevents the myocardium from maladaptive hypertrophy, fibrosis and cardiomyocytes apoptosis, reducing cardiac dysfunction and improving survival [6]. Since sST2 functions as a soluble decoy receptor of IL-33, it attenuates the protective effects of the IL-33/ST2L signaling pathway and works in a dose dependent manner [13]. sST2 was also identified to promote the processes of healing, myocardial fibrosis and cardiac remodeling [31].

Inflammation has been recognized to play an important role in the pathogenesis of different cardiovascular diseases. Higher concentrations of sST2 were associated with disease progression and predicted prognosis. As its concentration is less prone to haemodynamic fluctuations, it may mirror the progress of myocardial remodeling [32]. Since ventricular remodeling is often involved in various kinds of heart failure, the modulating role of sST2 in the IL-33/ST2L signaling pathway reflects its potential value as a biomarker for heart failure. In addition to its role as a biomarker of myocardial fibrosis and remodeling, multiple studies have suggested sST2 to be one of the most powerful prognostic biomarkers in both acute and chronic heart failure [11], [33]. The relationship of sST2 concentrations and mortality is reportedly independent of other relevant clinical and biochemical parameters [34]. There appears to be a dose–response relationship between sST2 concentration and such risk, consistent with the notion that its molecular action in IL-33 signaling is itself dose-dependent [13].

## 5 Strengths and limitations

This study has many strengths. Firstly, all included studies had a prospective design, so our results are more accurate and less prone to biases. Secondly, most of the included studies used multivariate analysis, reducing the influence of confounders. However, several limitations should be acknowledged. There was a high degree of heterogeneity observed. This may be due to differences in quantification assays used for sST2 and country of origin. Despite the promising predictive value of sST2 in predicting mortality, we did not define a specific cut-off value due to the heterogeneity among studies and selection criteria. Whilst the goal of this *meta*-analysis was to examine possible associations between sST2 levels and mortality in different cardiovascular conditions, it did not aim to provide risk quantification. Moreover, it did not examine sensitivities and specificities of sST2, which would be important for assessing its diagnostic ability. Future studies can perform *meta*-analysis of these statistical measures to explore this further. Therefore, the value of sST2 should be interpreted in the clinical context of the patient in conjunction with other laboratory test results for the purposes of risk stratification and deciding appropriate management.

## 6 Conclusion

High sST2 levels predict mortality in chronic heart failure and in stable coronary heart disease. It therefore provides incremental value for risk stratification purposes in these patient populations.

## Declaration of Competing Interest

The authors declare that they have no known competing financial interests or personal relationships that could have appeared to influence the work reported in this paper.
