# Supplementary material for: Soluble suppression of tumorigenicity 2 (sST2) for predicting disease severity or mortality outcomes in cardiovascular diseases: A systematic review and meta-analysis
Source: Int J Cardiol Heart Vasc. 2021 Oct 18;37:100887. doi: 10.1016/j.ijcha.2021.100887 (PMC8528731; doi:10.1016/j.ijcha.2021.100887)
Supplement: Supplementary data 1 [file mmc1.docx]

**Supplementary Figures**

**
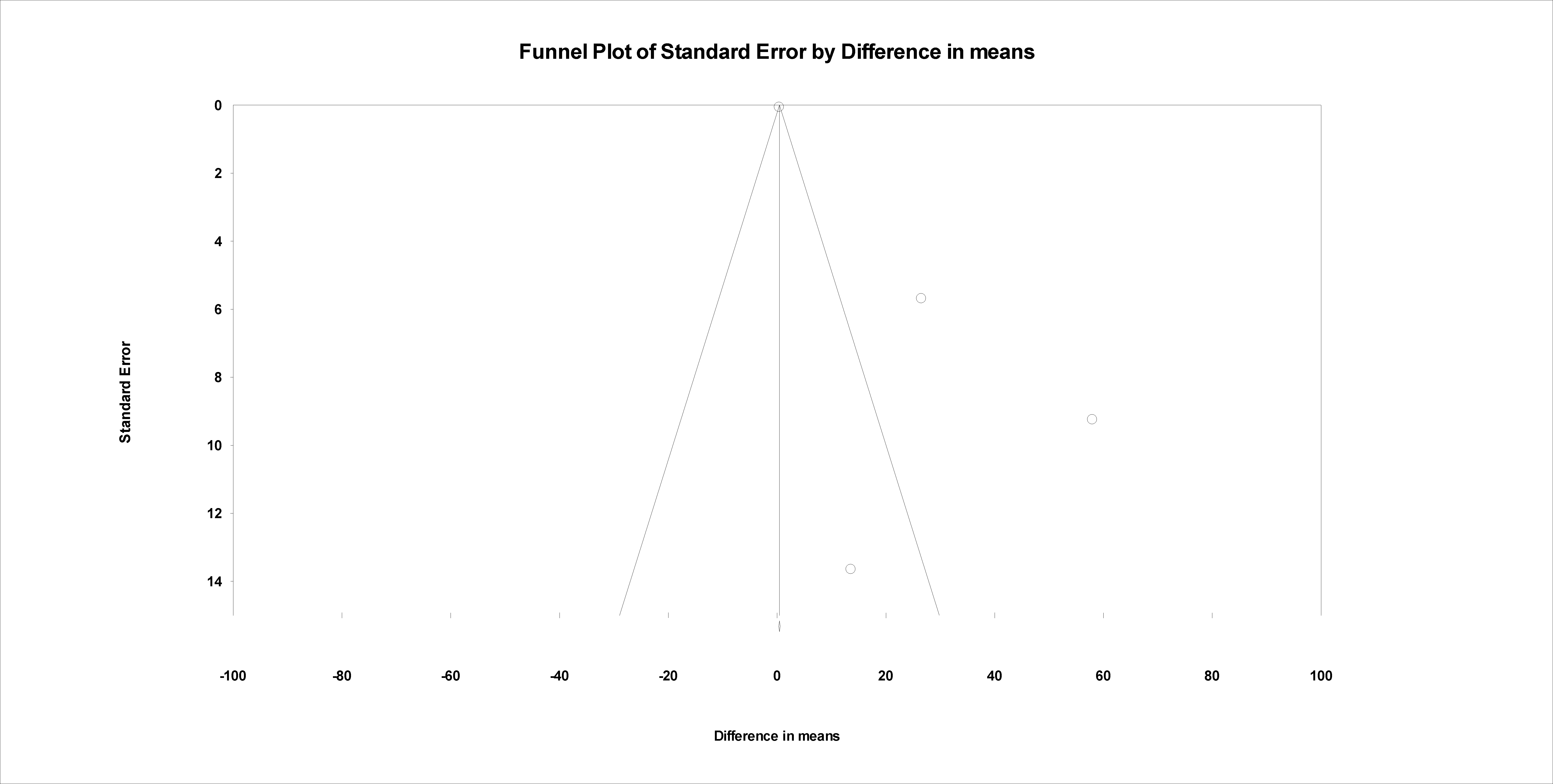
**

**Supplementary Figure 1.** Funnel plot of standard errors against differences in means in sST2 between non-survivors and survivors in acute heart failure.

**
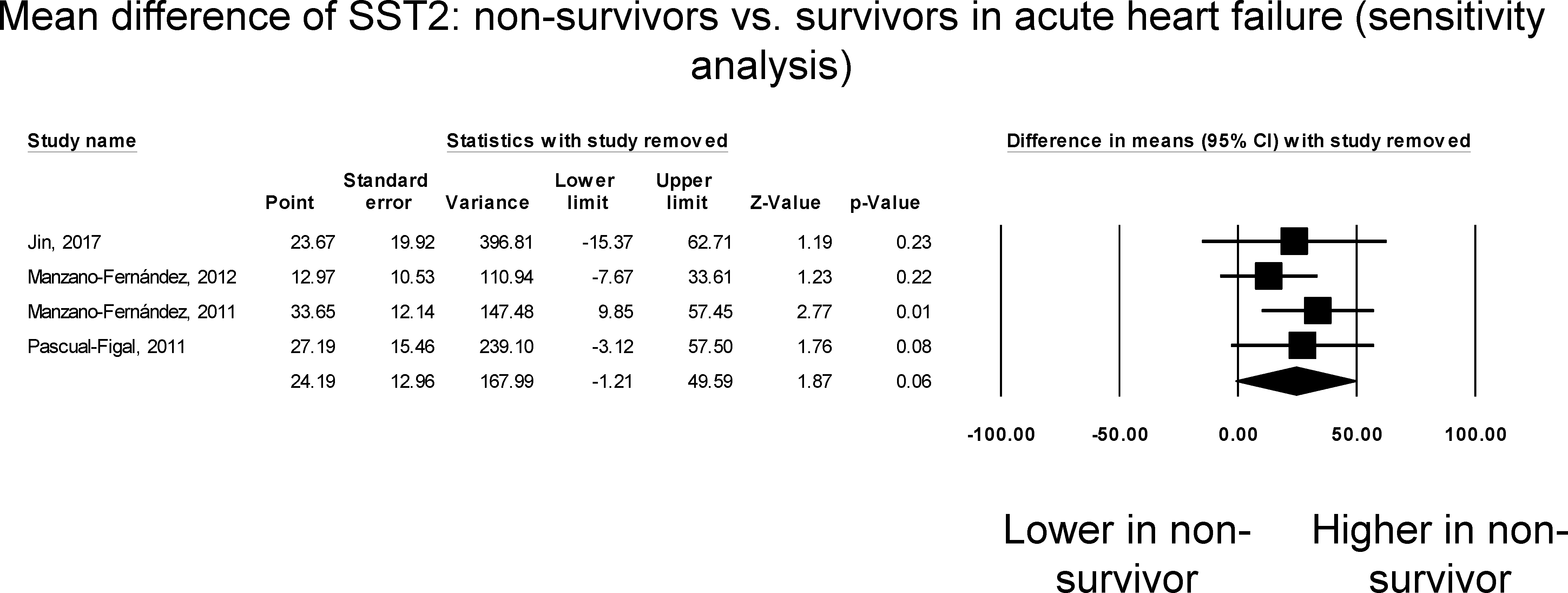
**

**Supplementary Figure 2.** Sensitivity analysis for mean difference of sST2 between non-survivors and survivors in acute heart failure.

**
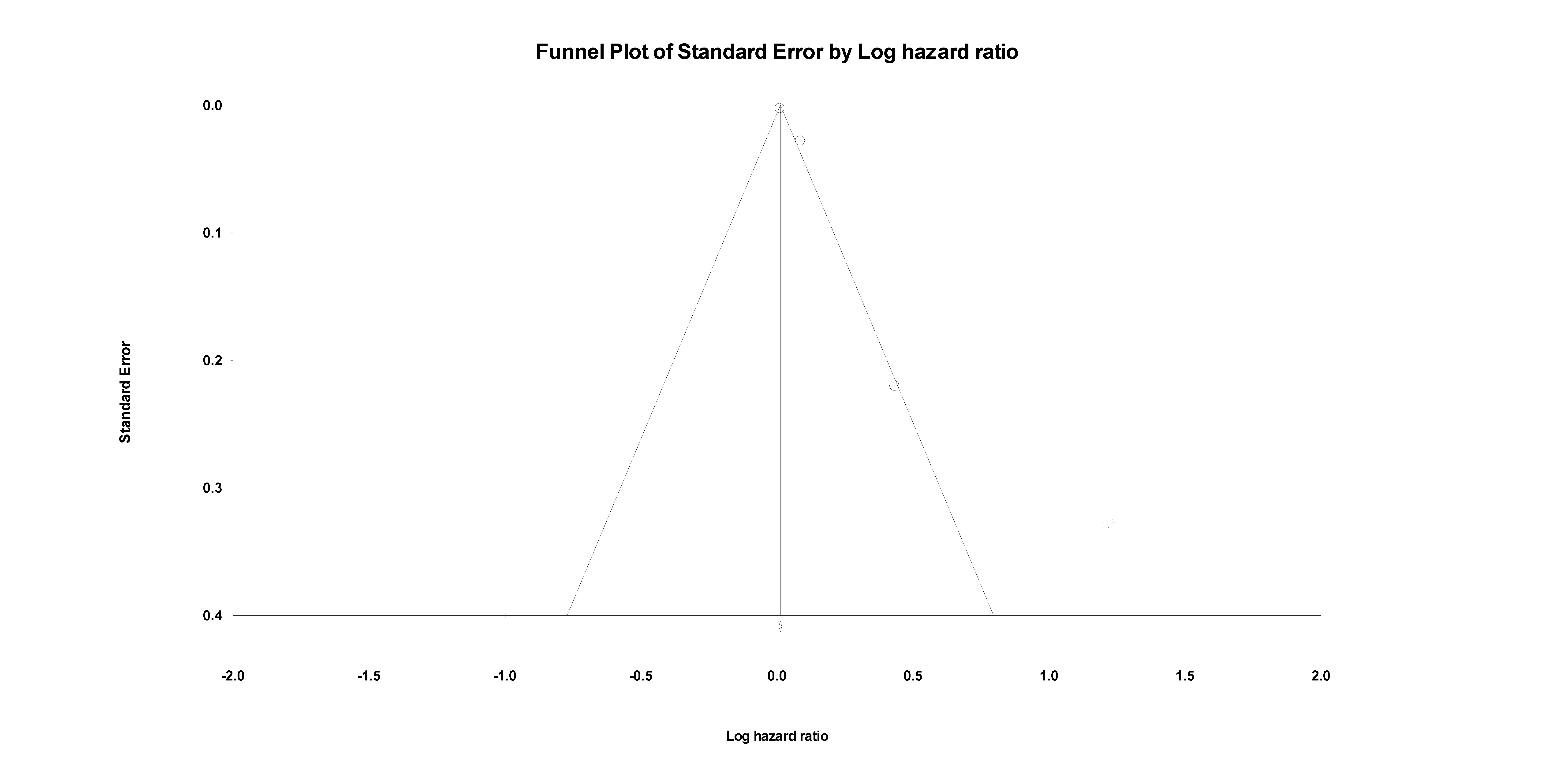
**

**Supplementary Figure 3.** Funnel plot of standard errors against logarithm of hazard ratios for mortality with high sST2 in acute heart failure.

**
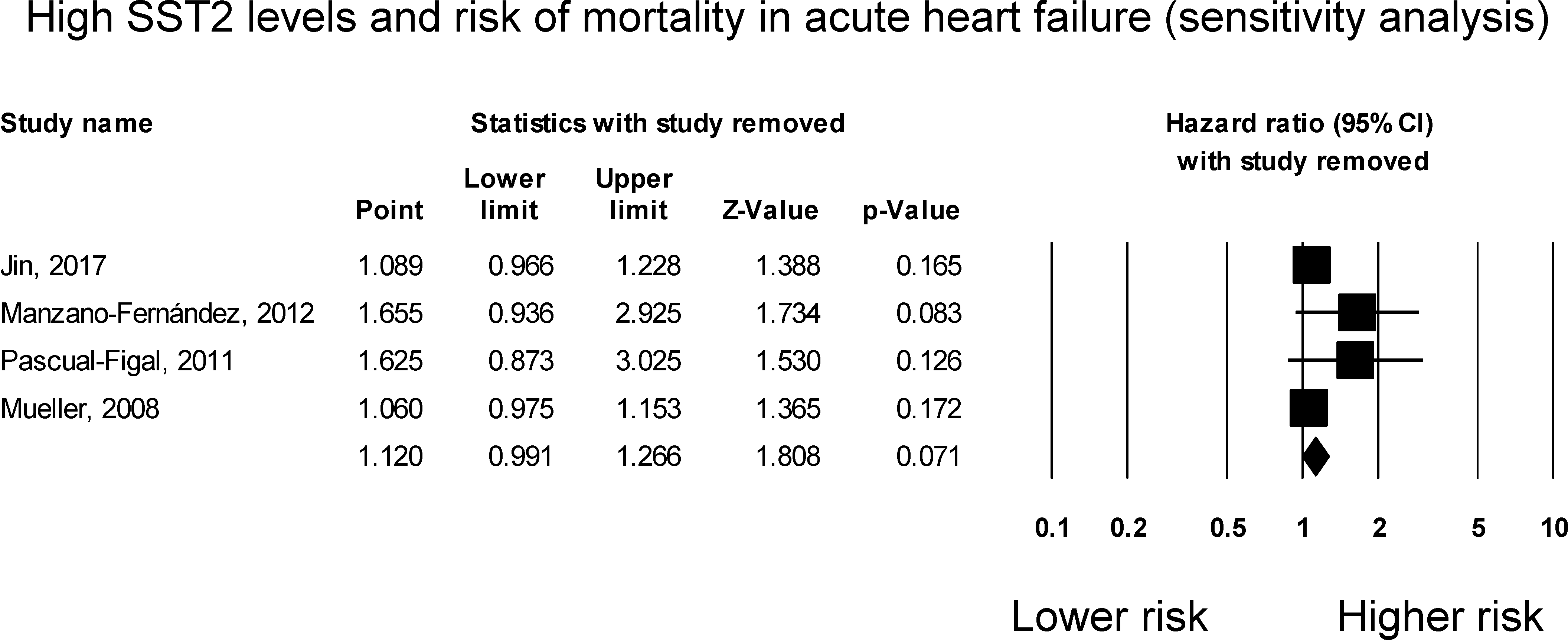
**

**Supplementary Figure 4.** Sensitivity analysis for hazard ratios for mortality with high sST2 in acute heart failure.


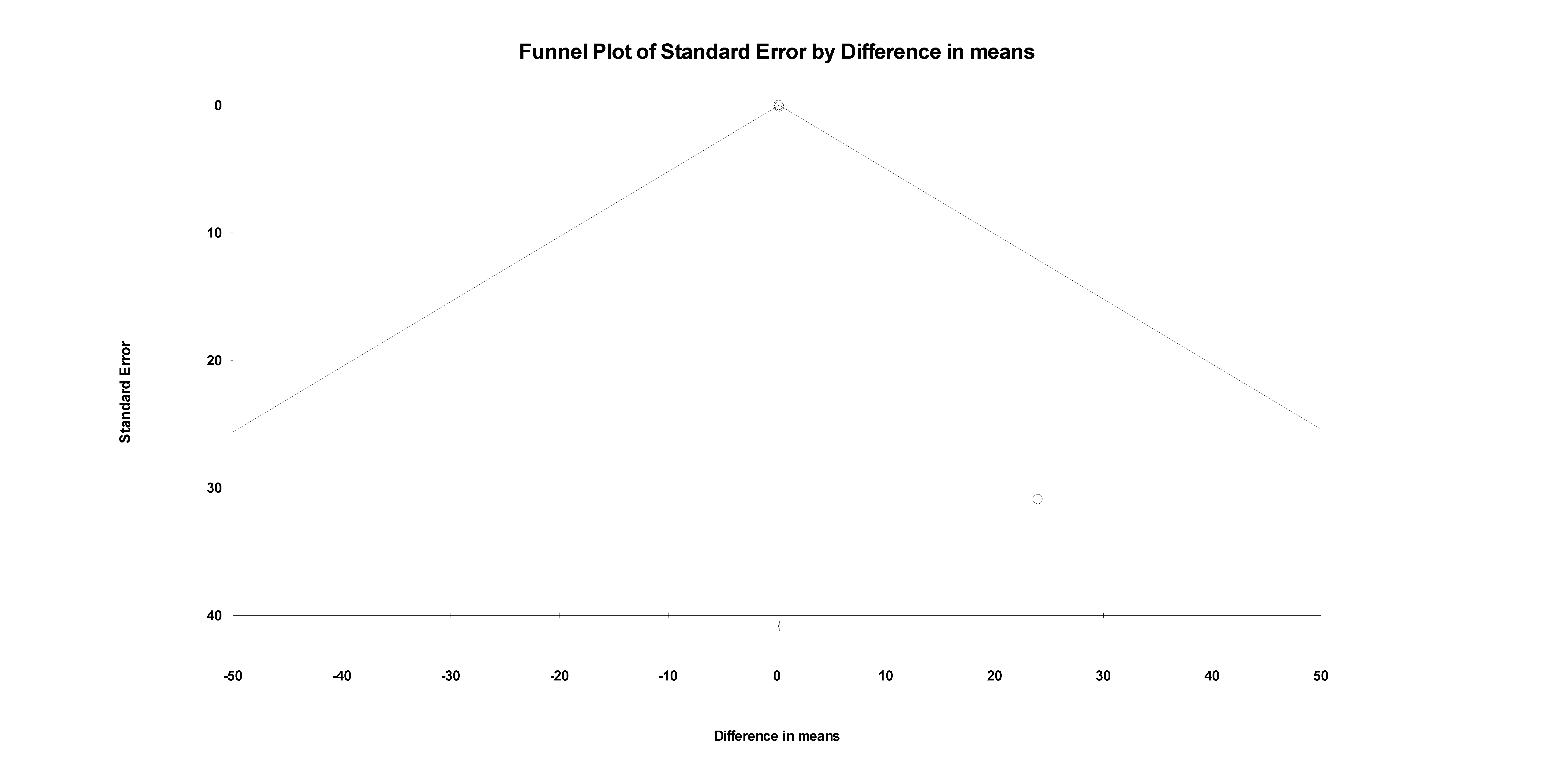


**Supplementary Figure 5.** Funnel plot of standard errors against differences in means in sST2 between non-survivors and survivors in chronic heart failure.

**
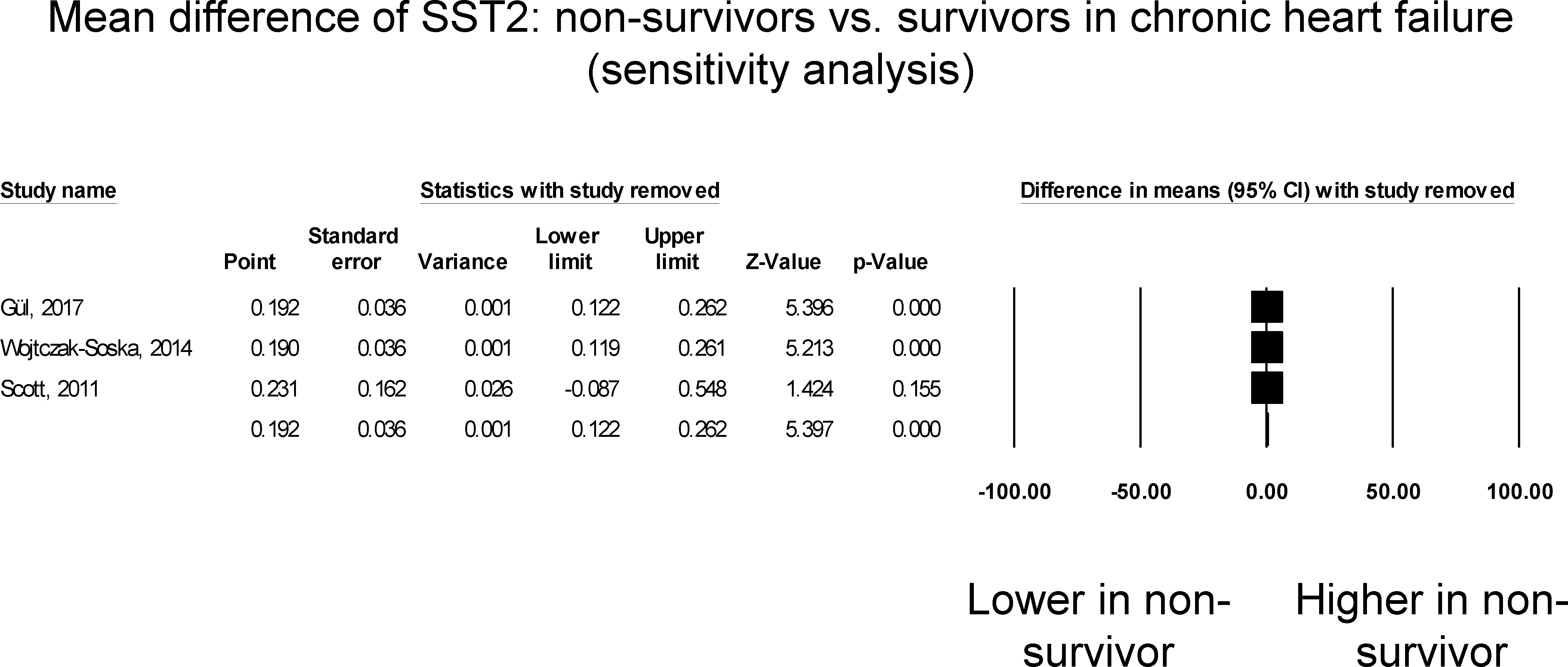
**

**Supplementary Figure 6.** Sensitivity analysis for mean difference of sST2 between non-survivors and survivors in chronic heart failure.

**
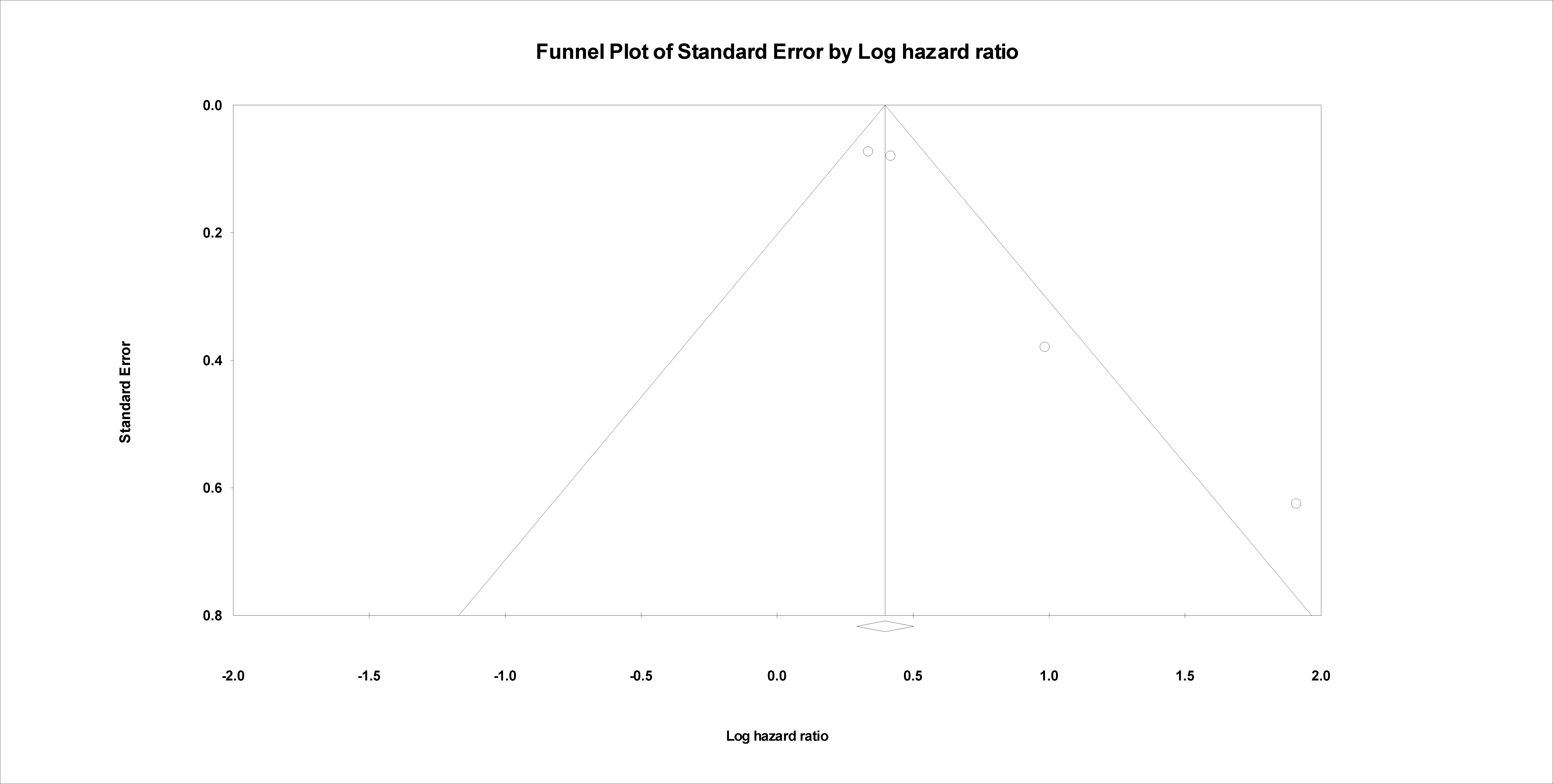
**

**Supplementary Figure 7.** Funnel plot of standard errors against logarithm of hazard ratios for mortality with high sST2 in chronic heart failure.

**
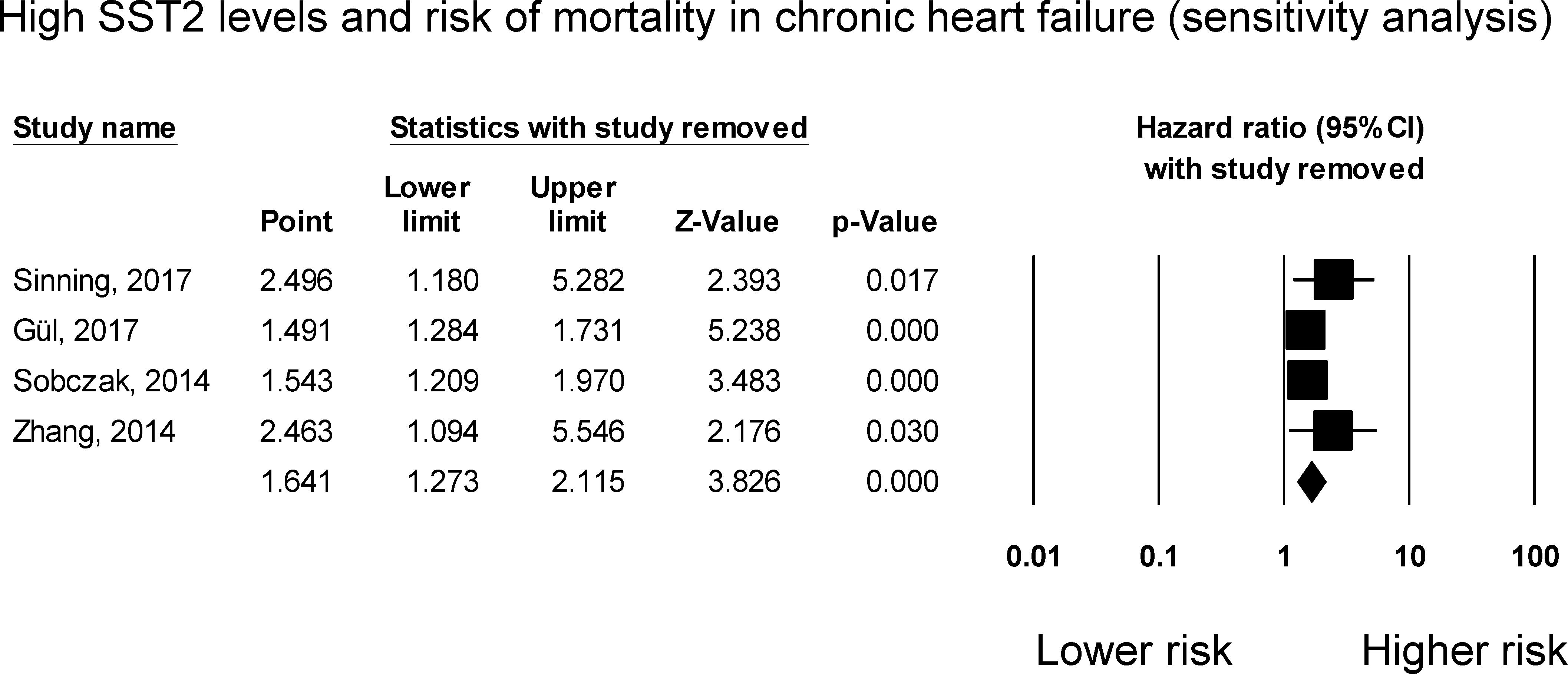
**

**Supplementary Figure 8.** Sensitivity analysis for hazard ratios for mortality with high sST2 in chronic heart failure.

**
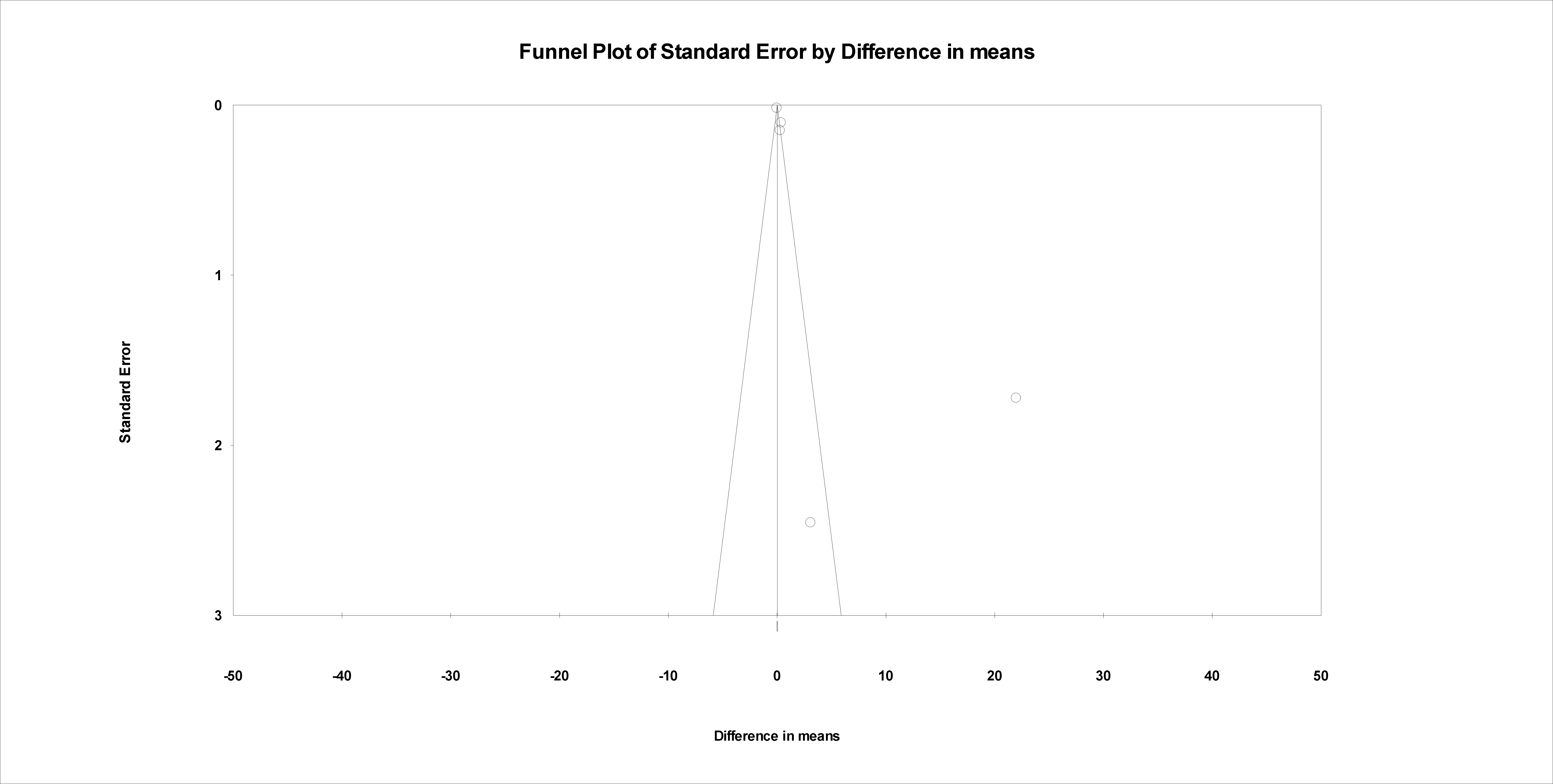
**

**Supplementary Figure 9.** Funnel plot of standard errors against differences in means in sST2 between severe disease and less severe disease in chronic heart failure.

**
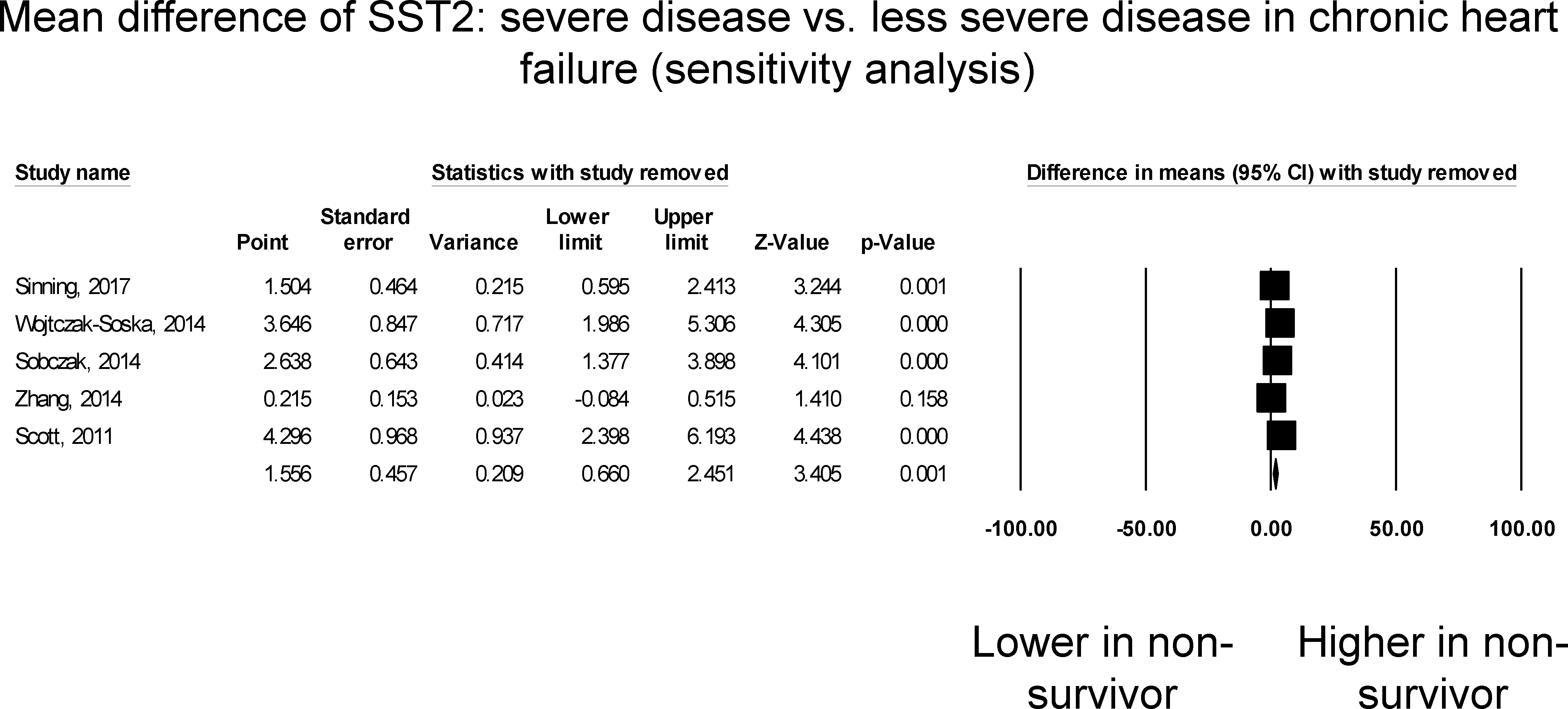
**

**Supplementary Figure 10.** Sensitivity analysis for mean difference of sST2 between severe disease and less severe disease in chronic heart failure.

**
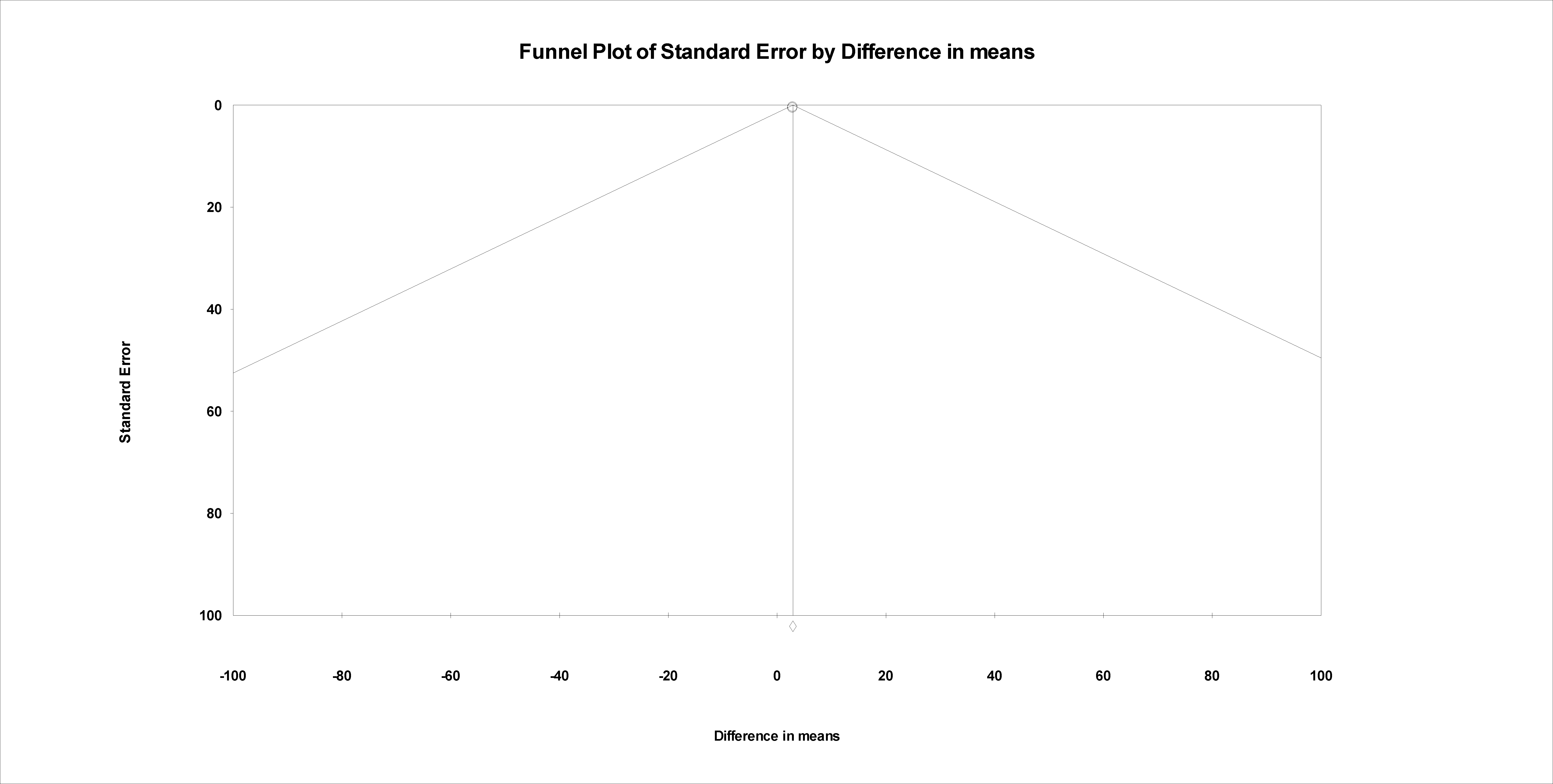
**

**Supplementary Figure 11.** Funnel plot of standard errors against differences in means in sST2 between non-survivors and survivors in stable coronary artery disease.

**
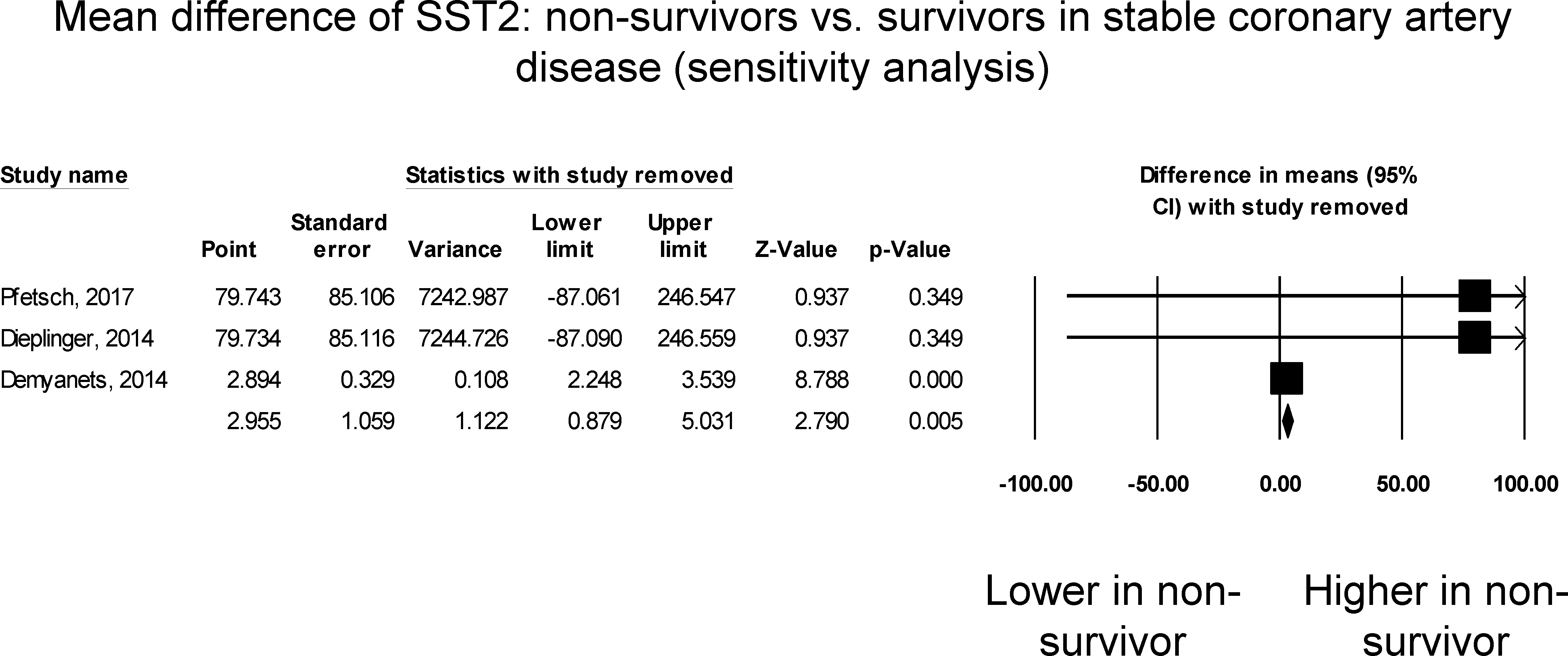
**

**Supplementary Figure 12.** Sensitivity analysis for mean difference of sST2 between non-survivors and survivors in stable coronary artery disease.

**
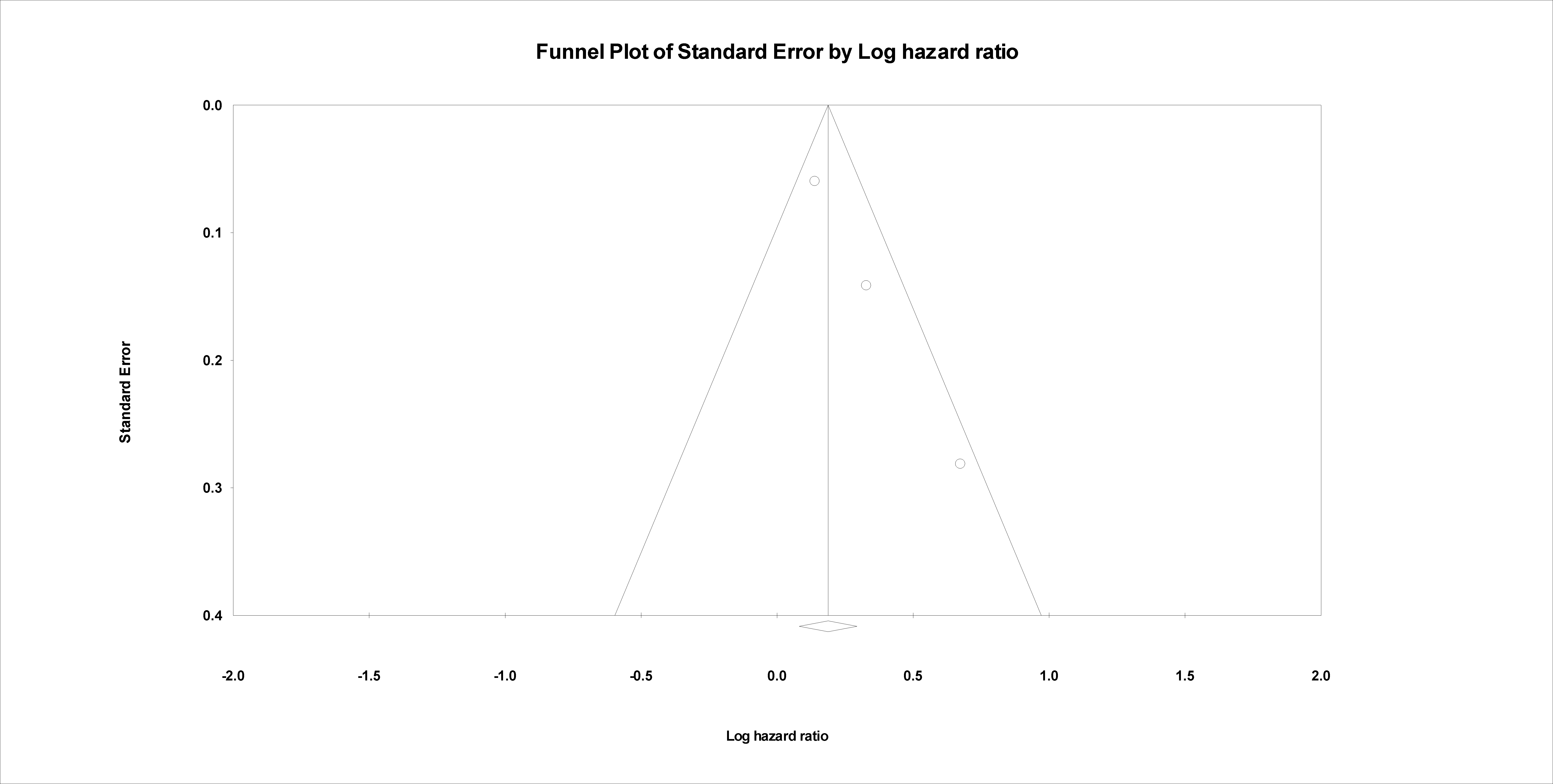
**

**Supplementary Figure 13.** Funnel plot of standard errors against logarithm of hazard ratios for mortality with high sST2 in stable coronary artery disease.

**
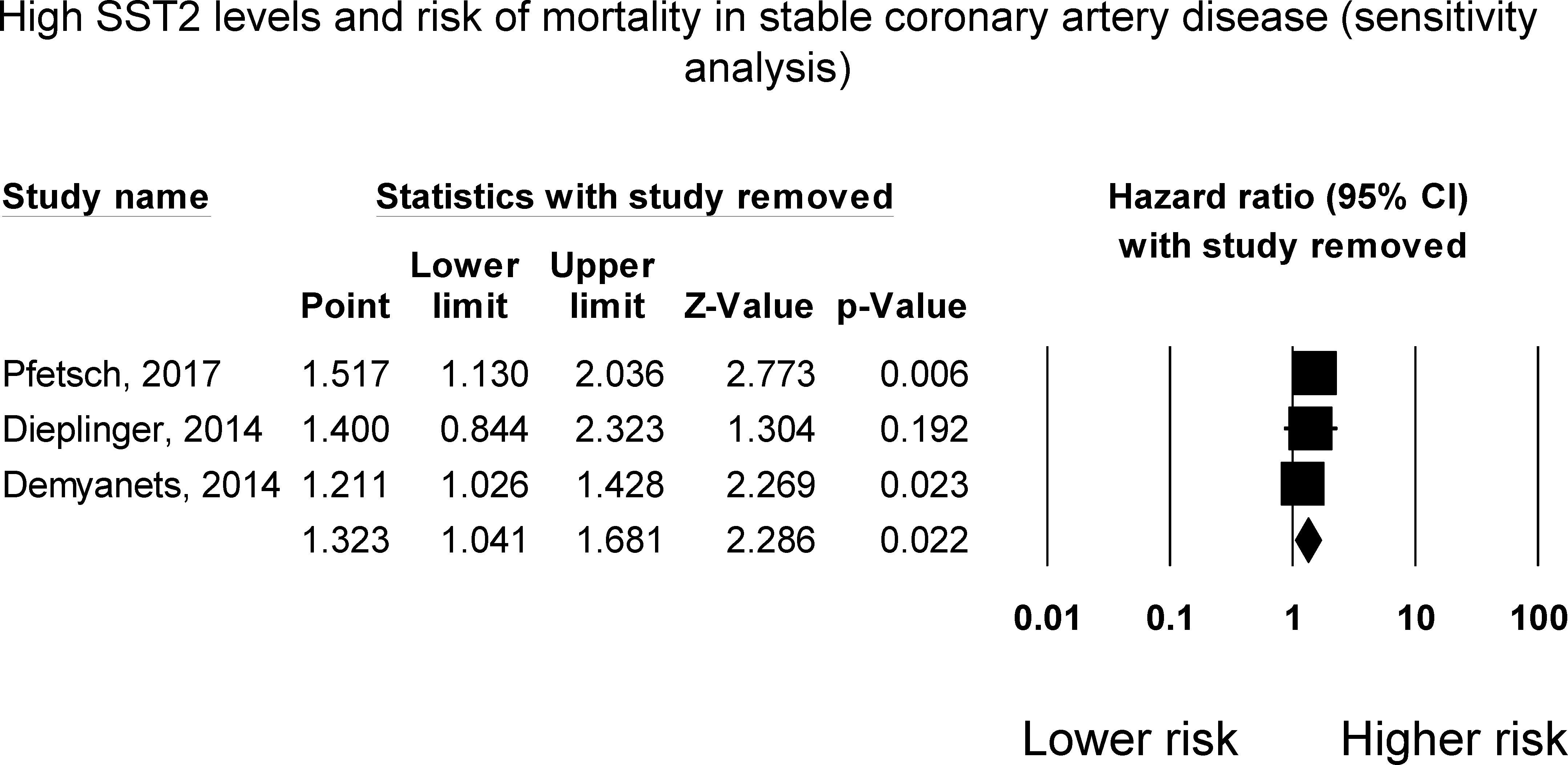
**

**Supplementary Figure 14.** Sensitivity analysis for hazard ratios for mortality with high sST2 in stable coronary artery disease.
