# Supplementary material for: Soluble suppression of tumorigenicity 2 (sST2) for predicting disease severity or mortality outcomes in cardiovascular diseases: A systematic review and meta-analysis
Source: Int J Cardiol Heart Vasc. 2021 Oct 18;37:100887. doi: 10.1016/j.ijcha.2021.100887 (PMC8528731; doi:10.1016/j.ijcha.2021.100887)
Supplement: Supplementary data 2 [file mmc2.docx]

|  | Selection | | | | Comparability | Outcome | | | Total score  (0-9) |
| --- | --- | --- | --- | --- | --- | --- | --- | --- | --- |
| Studies | Representativeness of the exposed cohort | Selection of the non-exposed cohort | Ascertainment of exposure | Outcome of interest not present at start of study |  | Assessment of outcome | Adequacy of duration of follow-up | Adequacy of completeness of follow-up |  |
| Demyanets, 2014 | 1 | 0 | 1 | 1 | 1 | 1 | 1 | 1 | 7 |
| Dieplinger,  2014 | 1 | 0 | 1 | 1 | 2 | 1 | 1 | 1 | 8 |
| Gul, 2017 | 1 | 0 | 1 | 1 | 2 | 1 | 1 | 1 | 8 |
| Jin, 2017 | 1 | 0 | 1 | 1 | 1 | 1 | 1 | 1 | 7 |
| Manzano, 2011 | 1 | 0 | 1 | 1 | 2 | 1 | 1 | 1 | 8 |
| Manzano, 2012 | 1 | 0 | 1 | 1 | 1 | 1 | 1 | 1 | 7 |
| Mueller, 2008 | 1 | 0 | 1 | 1 | 1 | 1 | 1 | 1 | 7 |
| Pascual, 2011 | 1 | 0 | 1 | 1 | 2 | 1 | 1 | 1 | 8 |
| Pfetsch, 2017 | 1 | 0 | 1 | 1 | 2 | 1 | 1 | 1 | 8 |
| Scott, 2011 | 1 | 0 | 1 | 1 | 1 | 1 | 1 | 1 | 7 |
| Sinning, 2017 | 1 | 0 | 1 | 1 | 2 | 1 | 1 | 1 | 8 |
| Sobczak, 2014 | 1 | 0 | 1 | 1 | 2 | 1 | 1 | 1 | 8 |
| Wojtczak, 2014 | 1 | 0 | 1 | 1 | 1 | 1 | 1 | 1 | 7 |
| Zhang, 2014 | 1 | 0 | 1 | 1 | 2 | 1 | 1 | 1 | 8 |

**Supplementary Table 1.** Newcastle-Ottawa Score (NOS) quality assessment of included studies.
